# Colistin and polymyxin B: A re-emergence

**DOI:** 10.4103/0972-5229.56048

**Published:** 2009

**Authors:** Sachin Gupta, Deepak Govil, Prem N. Kakar, Om Prakash, Deep Arora, Shibani Das, Pradeep Govil, Ashima Malhotra

**Affiliations:** **From:** Department of Anaesthesiology, Max Super Speciality Hospital, 1 Press Enclave Road, Saket, New Delhi, India; 1 Department of Critical Care, Artemis Health Institute, Sector-51, Gurgaon 122 001, Haryana, India

**Keywords:** Multidrug-resistant organisms, polymyxins

## Abstract

One of the greatest achievements of modern medicine is the development of antibiotics against life-threatening infections, but the emergence of multidrug-resistant (MDR) gram negative bacteria has drastically narrowed down the therapeutic options against them. This limitation has led clinicians to reappraise the clinical application of polymyxins, an old class of cationic, cyclic polypeptide antibiotics. Polymyxins are active against selected gram-negative bacteria, including the *Acinetobacter* species, *Pseudomonas aeruginosa, Klebsiella* species, and *Enterobacter* species. In this article, we summarise the chemistry, pharmacokinetics, and pharmacodynamics of polymyxins and the latest understanding of their action against MDR pathogens.

## Introduction

Globally, there is a growing threat from the emergence of multidrug-resistant organisms[1–3] especially gram-negative bacteria, such as *Pseudomonas aeruginosa, Acinetobacter baumannii,* and *Klebsiella* spp. Mortality, morbidity, and health care costs are substantially increased by infections caused by these agents.[4,5]

Polymyxins, a group of cationic polypeptide antibiotics consisting of 5 chemically different compounds (Polymyxins A-E), were discovered in 1947.[6] Only polymyxin B and polymyxin E (colistin) have been used in clinical practice.

Colistin and polymyxin B are produced from *Bacillus* spp.[7] Colistin was synthesized non ribosomally from *Bacillus polymyxa* subspecies *colistinus*.[8] Soon after their introduction into clinical use in 1949, concerns arose about adverse effects (nephrotoxicity, ototoxicity, and neuromuscular blockade) associated with their use. The recent antibiotic therapeutic void against gram-negative organisms has rapidly evolved into a modern polymyxin era since the late 1990s.

## Clinical pharmacology

### Chemical structure

Colistin is a cationic, multicomponent lipopeptide consisting of a cyclic heptapeptide with a tripeptide side chain acylated at the N terminus by a fatty acid. The two major components of colistin are colistin A (polymyxins E_1_) and colistin B (polymyxins E_2_).[9] Different pharmaceutical preparations of colistin may contain different amounts of these two components.

### Mechanism of action

Colistin and polymyxin B have their antimicrobial activity mainly directed against the bacterial cell membrane. The cationic polypeptides of colistin and polymyxin B interact with anionic lipopolysaccharide (LPS) molecules in the outer membrane of gram-negative bacteria, leading to displacement of calcium (Ca^2+^) and magnesium (Mg^2+^), which stabilize the LPS membrane, thus causing derangement of the cell membrane. This results in an increase in the permeability of the cell membrane, leakage of cell contents, and ultimately cell death.[10–12]

Colistin also has potent anti-endotoxin activity. The endotoxin of gram-negative organism is the lipid A portion of LPS molecule and colistin binds and neutralizes this LPS molecule.

### Pharmacokinetics

Colistin is available commercially in two forms: colistin sulfate, which is used topically and orally and CMS (colistimethate sodium, colistin methanesulfate, pentasodium colistimethanesulfate, and colistin sulfonyl methate) used for parenteral and inhalational use. CMS is an inactive prodrug of colistin[13] and is less potent and less toxic than colistin sulphate.[14] Polymyxin B and colistin differ only in their amino acid components. These antimicrobials are not absorbed by the gastrointestinal tract with oral administration. Colistimethate is not stable *in vitro*[15] or *in vivo*[16] and is hydrolysed into a complex mixture of partially sulfomethylated derivatives and colistin.

CMS and colistin differ in their pharmacokinetics. CMS is excreted primarily by the kidney and it involves renal tubular secretion. On the other hand, colistin is mainly excreted by non renal mechanisms that are as yet not fully understood. In renal impairment, the excretion of colistimethate by the kidney is decreased and a greater fraction of CMS is converted to colistin; this explains the rationale to decrease the dose of CMS in patients with renal impairment who are not receiving renal replacement therapy.[17] Polymyxins concentrate in the liver, kidney, muscle, heart, and lungs but do not cross the blood-brain barrier in non inflammed meninges. Presently, there are no reliable pharmacokinetic models for polymyxin B that can generate the dose adjustments in patients with renal impairment. There are very few studies conducted with polymyxin B regarding its safety in renal failure, so the dose adjustments mentioned are based on the results of those studies as well as the recommendations by manufacturers.

### Pharmacodynamics

Most pharmacodynamic data on colistin are from in-vitro studies.[18,19] Both colistin and polymyxin B have potent, concentration-dependent killing capacity against MDR gram-negative bacteria as per the in-vitro time-kill studies.

In a recent study, Owen, *et al*.[20] discussed the relationship between the post antibiotic effect and the dosing duration of colistin. In view of the lack of sufficient post-antibiotic effects after treatment with CMS (the parenteral form of colistin), and the relatively short half-life of colistin (4 hours) after IV administration of CMS, once daily administration would not be a good option for treatment of the MDR *A. baumannii*. Therefore, the suggested monotherapy with CMS in MDR bacteria with long-dosing intervals may be problematic.

### Spectrum of activity

Colistin has excellent bactericidal activity against most gram-negative aerobic bacilli, including *Acinetobacter* species (MIC_90_ ≤ 2 mg/L), *P. aeruginosa* (MIC_90_ ≤ 4 mg/L),[21] *K. pneumoniae* (MIC_90_ ≤ 1 mg/L), *E. coli* (MIC_90_ ≤ 2 mg/L), and *Enterobacter* spp (MIC_50_ ≤ 1 mg/L). It also may be active against *Salmonella* spp (MIC_90_ ≤ 1 mg/L), *Shigella* spp (MIC_90_ ≤ 0.5 mg/L), *Citrobacter* spp (MIC_90_ ≤ 1 mg/L), *Yersinia pseudotuberculosis, Haemophilus influenzae,* and several mycobacterial species. *Providentia* spp, *Serratia* spp, *Brucella* spp are all resistant to colistin.[7,19]

## Dosage

Colistin is available commercially in two different formulations. Colomycin (Dumex-Alpharma, Denmark) has half million, 1 million or 2 million IU per vial[17] i.e., about 12,500 units/mg whereas Coly--Mycin M parenteral (Parkedale Pharmaceuticals, USA) contains 150 mg colistin base activity. The recommended dosing schedule varies as per the manufacturer. Colomycin manufacturers recommend 50,000 to 75,000 IU/kg/day (4–6 mg/kg/day of colistin) in three divided doses. On the other hand, Coly--Mycin manufacturers recommend a dose of 2.5 to 5 mg/kg colistin base activity per day in two to four divided doses (6.67-13.3 mg/kg/day of CMS).[22] Various studies support an acceptable safe dose of 6–9 mg/kg/day of Colomycin[23] and 2.5–5 mg/kg/day (6.67-13.3 mg/kg/day) with Coly--Mycin. So there is a possibility of under-dosing with colomycin if it is administered in the recommended doses of manufacturers. Colistin is available in India as Xylistin (Cipla Pharmaceuticals), containing half million or 1 million units per vial and the dose followed is 50,000 to 75,000 IU/kg/day.

The recommended dosage of intravenous polymyxin B is 1.5 to 2.5 mg/kg/day (1 mg = 10,000 IU) divided in two equal doses.

In patients with renal impairment, the dosage of colistin and polymyxin B needs modification. For a serum creatinine level of 1.3 to 1.5 mg/dL, 1.6 to 2.5 mg/dL, or above 2.6 mg/dL, the recommended dosage of IV colistin is 160 mg (2 million IU) every 12 hours, 24 hours, or 36 hours, respectively. For patients undergoing hemodialysis, the dosage of colistin is 80 mg (1 million IU) after each hemodialysis treatment. A dose adjustment of polymyxin B in renal failure has not been well established but one such dose adjustment is based on creatinine clearance (CrCL). If CrCL is 20 to 50 mL/minute, administer 75% to 100% of the normal daily dose in divided doses every 12 hours. If CrCL is between 5 to 20 mL/minute, administer 50% of the normal daily dose in divided doses every 12 hours. If CrCL is <5 mL/minute, administer 15% of the normal daily dose in divided doses every 12 hours.[24]

Aerosolised colistin in a dosage of 80 mg/day (1 million IU) for patients ≤ 40 kg and 160 mg/day (2 million IU) for patients > 40 kg are being used as an adjunctive intervention in treatment for MDR pneumonia. Bronchoconstriction, cough, and chest tightness have been reported during colistin nebulisation.[25]

Intraventricular or intrathecal administration of colistin may prove to be a life-saving intervention for patients with meningitis caused by MDR gram-negative organisms not responding to intravenous colistin.[26–28] Various case reports support this route of administration and according to these studies, the recommended dose is 3.75 to 10 mg colistin per day. Bukhary, *et al*.[27] treated a case of meningitis caused by MDR *P.aeruginosa* by an intrathecal route.

## Administration

### Intravenous

Dissolve 2 million units in 300 ml to 500 ml of 5% dextrose for a continuous intravenous drip. In patients with renal failure not on any renal replacement therapy, dissolve the 24-hour dose in 50 ml of normal saline and administer it as a 24-hour infusion via an infusion pump. The antibiotic can also be given at regular intervals by dividing the daily dosage into three or four equal doses and dissolving it in 100 ml 5% dextrose.

### Intramuscular

Not recommended as it is very painful at the injection site.

### Intrathecal

Dissolve half million units in 10 ml of sterile physiologic saline (Sodium Chloride Injection, USP) for a concentration of 50,000 units per ml.

### Inhalation

For patients breathing spontaneously, 80 mg (1 million U) of colistin is added to 4 ml of normal saline and nebulised with 8 lt/min oxygen flow via a face mask. For patients on mechanical ventilation, aerosolized colistin can be delivered just like other nebulisation.

## Toxicities

The most common potential toxicities with intravenous administration of polymyxins are nephrotoxicity and neurotoxicity.[14] Both of these toxicities are dose-dependent. Renal toxicity mainly includes acute tubular necrosis leading to an increase in serum urea and creatinine levels. Polymyxins can also lead to hematuria, proteinuria, and cylinduria. However, the high incidence of nephrotoxicity in earlier studies[29,30] was due to a lack of understanding of pharmacokinetics, pharmacodynamics, and inappropriate dosage schedules of polymyxins.[14] Recent investigations have revealed CMS to be less nephrotoxic than amikacin or tobramycin.[14]

The neurotoxic effects include oral and perioral paraesthesia, headache, ataxia, vertigo, visual disturbances, confusion, vasomotor instability, and reversible neuromuscular blockade. In the last decade, no case reports have reported the neurological side-effects of these antimicrobials.

Other miscellaneous side-effects are hypersensitivity reactions (contact dermatitis, rash, itching), fever, ototoxicity, and mild gastrointestinal disorders. Side-effects due to aerosolized administration of polymyxins are cough, bronchoconstriction, and chest tightness. A high-dose administration of intrathecal colistin may cause convulsions.

## Resistance

The mechanism of resistance to the polymyxins remains poorly understood. Various studies have suggested that alterations of the outer membrane of bacterial cell-like loss of LPS,[31] reduction of specific outer membrane proteins, reduction in cell envelope Mg^2+^ and Ca^2+^ contents, and lipid alterations are related to the development of resistance.[32] Polymyxin B and E resistance mechanisms appear to be either stable (mutational) or reversible upon removal of selective pressure (adaptive). A few studies have suggested that heteroresistance to colistin is not as common in MDR *P. aeruginosa* as in MDR *A. baumanii*.

There is an urgent need to define appropriate dosing regimens for all populations of MDR organisms and especially patients with *A. baumanii* infections.

## Polymyxin use in a critical care setting

Polymyxin B and E is currently considered for the treatment of MDR gram-negative infections confirmed by *in vitro* susceptibility testing.

### Ventilator-associated pneumonia (VAP)

Most research work has been done to check the efficacy of colistin. The usefulness of colistin as a treatment option in ventilator-associated pneumonia has been done in many studies.

Garnacho-Montero, *et al.*[33] administered CMS intravenously to 21 of 35 patients with VAP due to MDR *A. baumanii* strains and reported a cure in 57% of the patients. Reina, *et al.*[34] examined the effectiveness and safety profile of colistin in infections caused by *A. baumanii* and *P. aeruginosa* in the ICU. There was no significant difference in cure rates and the adverse effect profile when compared with the carbepenem group. Studies by Levin,[35] Linden,[36] and Markou[23] in patients with VAP due to *A. baumanii* and *P. aeruginosa* confirmed the equal efficacy of intravenous colistin with carbepenems.

Michalopoulus, *et al.*[37] used aerosolized colistin for the treatment of MDR gram-negative nosocomial pneumonia in patients without cystic fibrosis. All the patients also received intravenous therapy concomitantly. A total of 7 out of 8 patients recovered, thus they concluded that aerosolised colistin can be used as a beneficial adjunctive therapy in the treatment of MDR VAP.

### Bacteremia/sepsis

Markou, *et al.*[23] administered colistin intravenously to patients with sepsis due to multiple causes. He observed a clinical response rate of 73% with 14.4% of the patients showing some deterioration in renal functions. Michalopoulus, *et al.*[37] studied the effect of colistin in sepsis due to MDR gram-negative organisms. He showed a clinical cure rate of 70%.

### Meningitis

Bukhary, *et al.*[27] treated a case of nosocomial MDR *A. baumanii* meningitis by administering 125,000 IU every 12 hrs in 5 ml normal saline intrathecally. There were no apparent side-effects. Berlana, *et al.*[38] treated 92% of patients with meningitis by administering colistin intravenously, intramuscularly, intrathecally, or inhaled. Similarly, Kasiakou, *et al.*[26] treated an isolated case of MDR meningitis by intraventricular administration of colistin.

### Combination therapy with colistin

There are very few studies that report the efficacy of combination therapy of colistin with other antimicrobials. A few studies have shown the synergistic activity of colistin with other antipseudomonal antibiotics like carbepenems, piperacillin/tazobactam, ceftazidime, or ciprofloxacin. Colistin and rifampicin have shown some synergistic activity against MDR strains of *A. baumanii*.

## Conclusion

Because there is little information on the pharmacokinetic and pharmacodynamic properties of colistin and polymyxin B, its appropriate use has been hampered. These agents have been recently used for MDR gram-negative organisms mostly *P. aeruginosa* and *A. baumannii* responsible for pneumonia, bacteremia, and urinary tract infections. The dosage used should be 160 to 240mg (2 to 3 million IU) per day for life-threatening infections and these doses should be adjusted according to the renal functions. Inhalational therapy with colistin or polymyxin B acts as adjunctive therapy in MDR gram-negative pneumonia.
